# Emulating tightly bound electrons in crystalline solids using mechanical waves

**DOI:** 10.1038/s41598-020-67108-0

**Published:** 2020-06-23

**Authors:** F. Ramírez-Ramírez, E. Flores-Olmedo, G. Báez, E. Sadurní, R. A. Méndez-Sánchez

**Affiliations:** 1 Posgrado en Ciencias e Ingeniería, División de Ciencias Básicas e Ingeniería, Universidad Autónoma Metropolitana-Azcapotzalco, Av. San Pablo 180, Col. Reynosa Tamaulipas, 02200 Ciudad de México, Mexico; 2 Departamento de Ciencias Básicas, Universidad Autónoma Metropolitana-Azcapotzalco, Av. San Pablo 180, Col. Reynosa Tamaulipas, 02200 Ciudad de México, Mexico; 3 Instituto de Física, Benemérita Universidad Autónoma de Puebla, Apartado Postal J-48, 72570 Puebla, Mexico; 4 Instituto de Ciencias Físicas, Universidad Nacional Autónoma de México, Apartado Postal 48-3, 62210 Cuernavaca Mor., Mexico

**Keywords:** Electronic properties and materials, Mechanical properties

## Abstract

Solid state physics deals with systems composed of atoms with strongly bound electrons. The tunneling probability of each electron is determined by interactions that typically extend to neighboring sites, as their corresponding wave amplitudes decay rapidly away from an isolated atomic core. This kind of description is essential in condensed-matter physics, and it rules the electronic transport properties of metals, insulators and many other solid-state systems. The corresponding phenomenology is well captured by tight-binding models, where the electronic band structure emerges from atomic orbitals of isolated atoms plus their coupling to neighboring sites in a crystal. In this work, a mechanical system that emulates dynamically a quantum tightly bound electron is built. This is done by connecting mechanical resonators via locally periodic aluminum bars acting as couplers. When the frequency of a particular resonator lies within the frequency gap of a coupler, the vibrational wave amplitude imitates a bound electron orbital. The localization of the wave at the resonator site and its exponential decay along the coupler are experimentally verified. The quantum dynamical tight-binding model and frequency measurements in mechanical structures show an excellent agreement. Some applications in atomic and condensed matter physics are suggested.

## Introduction

The determination of the electronic band structure in crystals is one of the most important problems in solid-state physics. Fortunately, in many cases, the electrons of a crystal are strongly attached to the atoms in the grid and consequently the band structure can be calculated easily. This fact is captured by the tight-binding (TB) model in which a very weak interaction with the neighboring atoms is assumed^1,2^. In the simplest picture of solids, the electronic wave functions of interacting atoms are expanded in terms of wave functions of isolated atoms, i.e. individual atomic orbitals. When the atomic nuclei are located at the sites of a periodic mesh, the corresponding expansion coefficients are given by discrete plane waves of quasi-momentum *k*, in compliance with Bloch’s theorem. It is important to note that this expansion obeys exclusively the symmetry of the atomic array, and does not incorporate any detail of the coupling between sites. However, when the interaction sets in, only the plane wave expansion above can be associated to a well-defined energy state: the wave is delocalized throughout the array (the electron does not belong to any particular site) and it determines the energy band structure by means of a dispersion relation *E*(*k*). The nearest-neighbour TB is then a particular case when the electron can “hop” only between nearest sites and the emergent bandwidth is proportional to the hopping energy or coupling. This model gives good quantitative results in many cases and can be improved with other methods when the TB model is not entirely satisfactory. Interactions with second and higher-order neighbours can also be included. The TB model offers the possibility of understanding metals, insulators, magnets and superconductors^3^. This model has also been considered as an ideal platform to explore emergent properties of novel materials, such as graphene^4,5^, hexagonal boron nitride^6^, stanene, germanene, silicene, among many others^7^. Furthermore it can be used to study molecules^8^, 2D electron gases^9^ and Bose-Einstein condensates in optical lattices^10^.

The tight-binding model, in addition, has been applied in areas of classical waves to study the properties of photonic^11–15^ and phononic^16–21^ crystals. This model has also been emulated in top-table experiments with microwaves, either with resonators mimicking atomic orbitals^22^ or with evanescent modes in waveguides^23,24^. All these experiments with classical waves, however, have only a limited use in the context of quantum mechanical systems since they have their own physical constants, such as hopping amplitudes, a *sui generis* dispersion relation and, as a consequence, their own dynamics.

In the structured elastic systems presented here, opposite to the cases mentioned above, the site energies and couplings can be engineered to get a “quantum tight-binding result” with first and second neighbours at least. The results shown here represent a breakthrough in the area of structured elastic systems since the application of the tight-binding model in this area has remained open for exploration due to several difficulties. One of them is that the typical coupling between connected vibrating solids is very strong and thus has a long range; an elastic evanescent coupling was introduced only very recently^25^. Also the different kinds of elastic waves, as for instance transverse and longitudinal, are strongly coupled; the selective excitation or detection of these elastic waves has been solved also recently^26,27^.

**Figure 1:**
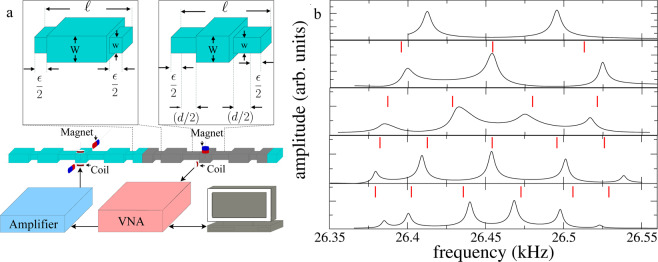
Schematic representation of the constructed system and the distribution of the frequency levels. (**a**) Bottom: Setup used to characterize the vibrations of the elastic structures with acoustic resonant spectroscopy. The setup is composed by a workstation, a vector network analyzer (Anritsu MB-4630B), a high-fidelity audio amplifier (Cerwin-Vega CV-2800), and two electromagnetic-acoustic transducers. Middle: the elastic structure to be characterized; it corresponds to two coupled resonators. The shadow zone indicate a supercell. Top left: 3D view of the unit cell of the coupler; it is composed by three cuboids: the central one with length $\ell -\epsilon$ = 92 mm, width and height *W* = 12.7 mm and two identical small cuboids, of length $\epsilon /2$ = 4 mm width and height $w=8.7$ mm, at both ends. Top right: a resonating cell, composed by three cuboids, is shown. The central cuboid has length $\ell -\epsilon -d$ = 55.2 mm, width and height *W* = 12.7 mm and two identical small cuboids, of length $(d+\epsilon )/2$ = 22.4 mm width and height *w* = 8.7 mm, at both ends. (**b**) From top to bottom measured spectra of the emergent band for the beams with 2, 3, 4, 5 and 6 supercells. The results of the tight-binding model are indicated by the vertical (red) lines.

In this paper, the emulation of evanescent (or weak) couplings *via* locally periodic structures is presented. In a similar fashion as the coupled-resonator optical waveguides are engineered with defects inside photonic crystals^11–15^, here coupled mechanical resonators are engineered in an otherwise periodic structure. The idea is to use the gaps of crystalline mechanical structures that will be taken as couplers. As it is well known, a periodic system, even an elastic one, shows bands and gaps. Therefore connecting neighbouring resonators through a locally periodic structure, allows them to communicate weakly with each other through the coupler when their resonant frequency lies within the coupler’s badgap. The maximum wave amplitude will be located at the position of the resonator and from there, the amplitude will decay exponentially through the coupler^25,28^. This result is used in order to build a mechanical metamaterial with transport through such *trapped states*. Here five mechanical vibrating systems, that obey the *quantum* tight-binding model and emulate a finite *quantum* 1D crystal, are reported. The tight-binding model here developed is a generalization to mechanical waves of the TB model that emerges for coupled-resonator optical waveguides^13,14^. In that model a set of periodic defects inside a photonic crystal are treated as high-Q resonators weakly coupled each other. The elastic systems were constructed on aluminum beams and are composed of *n* resonators joined by couplers (see Fig. 1). Each coupler is formed by *m* unit cells of length $\ell$. In turn, each one of these unit cells is composed of a large cuboid of cross-sectional area *W* × *W* and length ℓ − *ϵ* and two small cuboids of cross-sectional area *w* × *w* and length $\epsilon /2$ where *W*, *w*, $\epsilon \ll \ell$ (see Fig. 1a, left inset). The resonator also has length $\ell$, and is composed of one cuboid of cross-sectional area *W* × *W* and length $l-\epsilon -d$ and two small cuboids of cross-sectional area $w\times w$ and length $(d+\epsilon )/2$ (see right inset of Fig. 1a). Two couplers of *M*(>*m*) unit cells are used as terminators to avoid finite size coupler’s border effects. The vibrating system can also be understood as a crystal composed by *n* coupled supercells (see shadow zone in the beam of Fig. 1). Each system was constructed by machining a solid aluminum piece.

The experimental spectra of the structured rods, for torsional waves, corresponding to two up to six *artificial elastic atoms* or supercells are shown in Fig. 1b. These spectra were measured using acoustic resonant spectroscopy by exciting at the position of one resonator and detecting at the position of another resonator. In this figure, the spectra show the emergence of a band, located within the second bandgap of the coupler (See ref. ^25^), as the number of supercells increases. As it can be seen in this figure, the band emerges symmetrically approximately at 26450 Hz, *i*.*e*. at a resonant frequency of the spectrum of a single artificial elastic atom. Each band is formed by well resolved Breit-Wigner line shapes with a number of resonances given by the number of artificial elastic atoms. The width of the band is approximately 150 Hz and it is possible to notice that the level spacings at the borders of the band are smaller than the level spacings at the center of the band, in agreement with the energy spectrum of a 1D atomic crystal.

**Figure 2:**
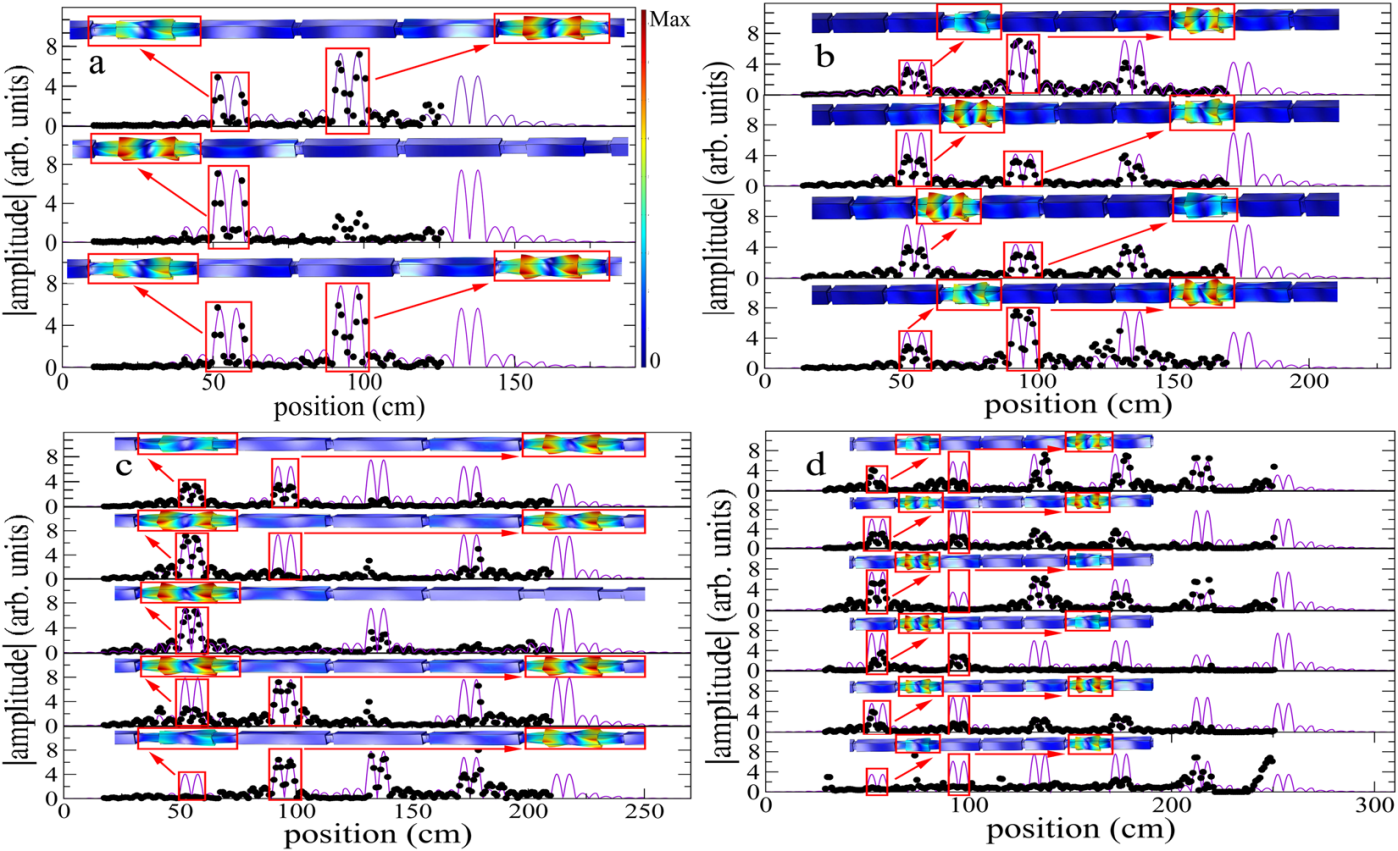
Experimental vs. FEM vs. TB model wave amplitudes. The absolute values of the torsional wave amplitudes, as function of the position, for the structured system with three, four, five and six coupled resonators are given in (**a**–**d**), respectively. The experimental and TB model results are given by dots and the continuous lines, respectively. In the upper part of each plot an amplification of the deformations of the elastic structure, obtained with finite elements, around two consecutive resonators, is shown. The color scale show the maxima (minima) of the deformations in red (blue). In descending order the amplitudes correspond to levels in the emergent band with frequencies (**a**) *f*_1_ = 26472 Hz, *f*_2_ = 26544 Hz and *f*_3_ = 26613 Hz. (**b**) *f*_1_ = 26461 Hz, *f*_2_ = 26512 Hz, *f*_3_ = 26574 Hz and *f*_4_ = 26622 Hz. (**c**) *f*_1_ = 26455 Hz, *f*_2_ = 26492 Hz, *f*_3_ = 26543 Hz, *f*_4_ = 26592 Hz and *f*_5_ = 26628 Hz, and (**d**) *f*_1_ = 26451 Hz, *f*_2_ = 26480 Hz, *f*_3_ = 26520 Hz, *f*_4_ = 26565 Hz, *f*_5_ = 26604 Hz and *f*_6_ = 26631 Hz, respectively.

In Fig. 2, top left (top right, bottom left, bottom right) the measurements of the absolute value of the torsional wave amplitudes, as a function of the position, for the elastic structure with three (four, five, six) coupled resonators are shown. Each wave amplitude has a one-to-one correspondence with each frequency level in the emergent band of Fig. 1. The obtained data were recorded by moving the detector along the beam; only two thirds of the total length of the structure were measured to avoid the saturation of the detector by the exciter’s magnetic field. Note that the experimental wave amplitudes are localized at the position of the resonators and that they show an exponential decay in the couplers. This is characteristic of states associated to frequencies in the bandgap and are compatible with the case of electrons strongly bounded to their atoms (tight-binding electrons). This fact is in agreement with the wave amplitudes obtained from the finite element numerical calculation in which the deformations of the structured beam can be seen mainly localized at the resonator positions.

**Figure 3:**
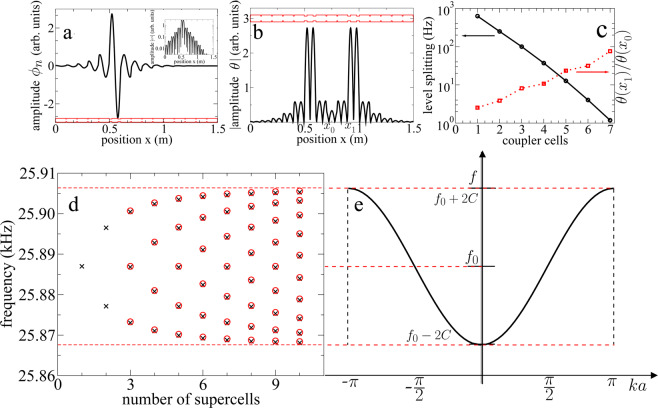
Wave amplitudes, level repulsion and dispersion relation of the emergent crystal. (**a**) Torsional wave amplitude ${\phi }_{n}(x)$, obtained with the transfer matrix method, as function of the position for a locally periodic beam with a resonator on site $n$. The longitudinal section of the elastic structure is shown in the lower part (red color). The exponential decay is shown in the inset. (**b**) Numerical wave amplitude $\theta (x)$ (absolute value) as a function of the position obtained with the transfer matrix method, for a a locally periodic beam with two coupled resonator. (**c**) Vertical left axis: level spacing as a function of the number of cells of the coupler. Vertical right axis: ratio $\theta ({x}_{1})/\theta ({x}_{0})$ where ${x}_{0}$ corresponds to the position central of the local maximum between both defects, while ${x}_{1}$ is the position of the global maximum. (**d**) Normal mode frequencies of the emergent band, as a function of the number of supercells, calculated with the tight-binding model (circles) and with the transfer matrix method (cross**e**s). (**e**) Dispersion relation of the mechanical crystal. The emergent band in **d** lies within the region limited by dispersion relation.

The experimental results given in Figs. 1 and 2 allow us to use the tight-binding approximation, *à la* quantum mechanics, to calculate the torsional spectrum of the elastic crystal. In other words, a mechanical system which can be described by the Anderson model is built. The basis of the mechanical TB model will be formed by the torsional wave amplitudes $\{{\phi }_{n}(x)\}$, localized at the position of the defect, with frequency *f*_*n*_ lying inside a gap of the coupler, associated to each isolated supercell on site $n$ (see Fig. 3a). The torsional wave amplitude $\theta (x)$ of the elastic crystal, as function of position, can be expanded in terms of the basis as

1 $$\theta (x)=\sum _{n}\,{A}_{n}{\phi }_{n}(x).$$

Then the tight-binding model for the 1D elastic crystal can be written as

2 $$-\,C{A}_{n-1}+{f}_{n}{A}_{n}-C{A}_{n+1}=f{A}_{n},$$

where, *f*_*n*_, is the resonant frequency of one isolated supercell on site *n* (Fig. 3a); *C* is the coupling coefficient between nearest neighbour resonators, which depends also on the properties of the coupler. The resonant frequency of the complete crystal will be *f*. This is the simplest TB model, since it assumes only nearest-neighbour couplings that are identical for all resonators. The generalization of this mechanism to more complex situations including second neighbours, as well as 2D and 3D systems, is straightforward. We should note that this model is mathematically equivalent to the quantum-mechanical and to the resonant-cavity coupled waveguide cases^13^, and it differs from the mass-spring tight-binding model (previously considered^29–31^), since the latter has ${\omega }^{2}$, instead of *f*, being $\omega$ the angular frequency. Thus *f*, *f*_*n*_, ${\phi }_{n}$ and *C*, in the TB model given in Eqs. (1) and (2) for mechanical waves, take the role of the energy, the site energy, the orbital function, and the hopping amplitude in the quantum TB model, respectively. The model represented in Eq. (2) has two free parameters, the site-frequency *f*_*n*_ and the mechanical hopping *C*. Physically *f*_*n*_ roughly corresponds to the resonant frequency of the isolated resonator (Fig. 3a) whereas *C* is related to the localization length of the wave amplitude of one resonator within the periodic structure. Both values should be determined to generate the TB torsional frequency spectrum for an elastic crystal with an arbitrary number of cells, as those given in Fig. 1. The values of *f*_*n*_ and *C* were obtained from the numerical calculations, made with the transfer matrix method, for a structure with two coupled resonators (Fig. 3b). In this case the TB model gives two solutions ${f}_{\pm }={f}_{0}\pm C$, thus giving the site-frequency *f*_0_ and the mechanical hopping *C* just as in the tight-binding model for the coupled-resonator optical waveguides^15^. Figure 3c gives the ratio *r* between the total maximum and the central local maximum of the symmetric wave amplitude of Fig. 3b, a measure related to the localization length for the two-resonators case, as a function the number of cells of the coupler. The level splitting Δ*f* = 2*C* is also given in Fig. 3c. As it can be seen in the same figure, the localization (level splitting) increases (decreases) as the number of cells in the coupler increases and that both quantities roughly show an exponential behaviour. One can also notice that the slope of the level splitting is approximately minus twice the slope of the ratio *r*, since the level repulsion can be understood as the overlap of two wave amplitudes ${\phi }_{i}$ of the basis located at different sites. In Fig. 3d the emergent band, appearing around 25.89 kHz, is given as function of number of supercells. As can be seen, the results obtained with the tight-binding model agree with those obtained with the transfer matrix method. It is possible to observe that the frequency levels split symmetrically around the level of frequency *f*_0_. This is in contrast with other elastic crystals studied in the literature^32^, whose levels are distributed asymmetrically towards one side of the first frequency level. This figure shows that the spectrum of the elastic crystal constructed here is completely analog to a spectrum of an 1D atomic crystal. In the mechanical case the resonators interact through the bandgap of the couplers. One might also consider an ideal elastic crystal, constructed from an infinite number of supercells. In this case it is possible to obtain the dispersion relation of the crystal. The wave amplitude on site *n* is written as a plane wave, ${A}_{n}={e}^{-inka}$, where $a$ is the distance between consecutive sites on the elastic lattice and *k* is the wave number. The dispersion relation

3 $$f={f}_{0}-2C\,\cos (ka),$$

is the same as that of a 1D periodic array of tightly bound electrons. This dispersion relation is shown in Fig. 3e; one can notice that the emergent band of the locally periodic system of *n* supercells, Fig. 3d, is completely contained inside the limits of the dispersion relation, ${f}_{0}\pm 2C$, of the ideal elastic crystal. The group velocity can be obtained directly from the dispersion relation and it implies that, at the bottom of the band, there are free mechanical quasiparticles analog to the free electrons in an 1D atomic crystal. In the mechanical case the quasiparticles have effective mass ${m}_{{\rm{eff}}}=\frac{1}{2C{a}^{2}}$.

In summary: in this article it was shown that it is possible to emulate tightly bound electrons in atomic crystals using mechanical waves. This was feasible thanks to the design of an elastic crystal composed of resonators joined with couplers in such a way that the levels associated with the resonators lie within the coupler’s bandgap. One should notice that the perturbative coupling used in refs. ^25,28,30–33^ is not enough to emulate a quantum tight-binding model; a resonator-coupler engineering using the Bragg reflection is needed. A generalization of resonant acoustic spectroscopy was used to measure the spectra and wave amplitudes of the artificial elastic lattices. The experimental results are well reproduced by the quantum tight-binding model. A complete analogy between the quantities used in the quantum model appear in the mechanical system: the on-site frequency, the mechanical hopping, the mechanical orbitals, the “frequency” levels, quasi-particles and an effective mass. The envelope of the atomic orbitals and the dispersion relation can also be obtained. The structured elastic systems reported here can be easily generalized to more complex quantum systems in 2D or 3D, such as molecules graphene, graphene nanoribbons with an electric field^34^ (changing the site size to emulate electric fields^33^), among many others. Transport through molecular junctions can also be implemented using the techniques recently developed by our group^35^. For obvious reasons, with the advantages and challenges of the macroscopic systems used, this gives the possibility of analyzing problems that cannot be studied easily in atomic systems due to experimental limitations. The mechanism described here allows to create mechanical metamaterials that have the features of a quantum crystal. Although the results presented are novel and have their value by themselves, they also open the door to the study of other quantum properties using mechanical waves. In fact, the effects of second neighbours on spectra and wave amplitudes can studied with the structured systems designed here. The previous statements are supported by the fact that coupling engineering in structured mechanical systems is flexible enough to introduce length deformations that generalize simple periodic configurations. The coupling engineering also allows to tune in the coupling with the distance since the model in the site basis is tridiagonal. The realization of evanescent couplings extends well beyond the mathematical abstraction of Wannier functions^36,37^ and the limitations of electronic transport in traditional solids.

## Methods

### Acoustic resonant spectroscopy

This technique is used to measure the mechanical vibration spectra for any elastic system and the setup is given in Fig. 1. The procedure starts with the generation of a harmonic signal, of frequency *f*_0_ in the vector network analyzer (VNA). This signal from the VNA is sent to a high-fidelity audio amplifier (Cerwin-Vega 2800 was used) to increase its power. The output of the amplifier is sent to an acoustic electromagnetic transducer (EMAT) located at the vicinity of the artificial elastic crystals. The EMAT, by electromagnetic induction generates mechanical vibrations in the aluminum piece^26^. A second EMAT detects the mechanical response of the crystal, at other location the beam, and converts it into a voltage signal. This signal is captured by the VNA. A workstation is used for the automated storage and subsequent analysis of the data of the different measurements. Then *f*_0_ is changed to *f*_0_ + Δ*f* and the procedure is repeated to obtain an spectrum. To excite and detect spectrum efficiently the transducers are located at the edges of the resonators. By moving the EMAT detector along the beam, it is possible measure the wave amplitudes as a function of the position.

### Electromagnetic acoustic transducers (EMATS)

We will first discuss how these devices excite mechanical waves (See Fig. 1). A harmonic current is applied to the EMAT coil and the latter produces a magnetic field, also alternating, which induces eddy currents in the metallic rod. The interaction, via Lorentz force, between the magnetic field of the magnet and the eddy currents produce a force on the metal. The same device can be used as a detector: when the metal surface oscillates close to the EMAT’s magnet, the magnetic flux through any loop of the paramagnetic metal will change. This, according to Faraday’s law, originates an electromotive force in the loops which in turn generates a magnetic field measured by the detector’s coil. A deeper explanation of the EMAT operation can be found in refs. ^27,33^.

### COMSOL simulations

COMSOL Multiphysics was used to calculate the finite element method simulations of Fig. 2 with the parameters corresponding to aluminum: Young’s module $E=68.6$ GPa, Poisson’s coefficient $\nu =0.33$ and density $\rho =2722$ kg m^−3^. Free boundary conditions were imposed and a symmetrical grid was used.

### Transfer Matrix

Lets consider a finite beam along the *z*-axis consisting of *M* cuboids of square cross section and side *w*_*i*_ with $i=1,2,\ldots ,M$. By definition the transfer matrix relates the amplitudes of the plane waves of $i$-cuboid with those of (1 + 1)-cuboid as

4 $$(\begin{array}{c}{A}_{i+1}\\ {B}_{i+1}\end{array})=\frac{1}{2}\left(\begin{array}{cc}\left(1+\frac{{w}_{i}^{4}}{{W}_{i+1}^{4}}\right){e}^{ik({z}_{i}-{z}_{i-1})} & \left(1-\frac{{w}_{i}^{4}}{{W}_{i+1}^{4}}\right){e}^{-ik({z}_{i}-{z}_{i-1})}\\ \left(1-\frac{{w}_{i}^{4}}{{W}_{i+1}^{4}}\right){e}^{ik({z}_{i}-{z}_{i-1})} & \left(1+\frac{{w}_{i}^{4}}{{W}_{i+1}^{4}}\right){e}^{-ik({z}_{i}-{z}_{i-1})}\end{array}\right)(\begin{array}{c}{A}_{i}\\ {B}_{i}\end{array}),$$

where the torsion in cuboid *i*, of width *w*_*i*_ (*W*_*i*_) and height *w*_*i*_ (*W*_*i*_) and located between positions *z*_*i*−1_ and *z*_*i*_, is ${\phi }_{i}(z)={A}_{i}{e}^{ik(z-{z}_{i-1})}+{B}_{i}{e}^{-ik(z-{z}_{i-1})}$. The continuity conditions for the torsion and the moment of torsion at $z={z}_{i}$ are ${{\phi }_{i}|}_{{z}_{i}}={{\phi }_{i+1}|}_{{z}_{i}}$ and ${{w}_{i}^{4}\frac{\partial {\phi }_{i}}{\partial z}|}_{{z}_{i}}={{W}_{i+1}^{4}\frac{\partial {\phi }_{i+1}}{\partial z}|}_{{z}_{i+1}}$. For a bar with square transversal section is given by $c=0.92\sqrt{\frac{G}{\rho }}$, where *G* is the shear modulus and $\rho$ is the density.

Defining the total transfer matrix as $T={T}_{M-1\to M}\cdot \cdot \cdot {T}_{i\to i+1}\cdot \cdot \cdot {T}_{1\to 2}$, the amplitudes at the right end of the beam can be written in terms of the amplitudes of the left end as

5 $$(\begin{array}{c}{A}_{M}\\ {B}_{M}\end{array})=T(\begin{array}{c}{A}_{1}\\ {B}_{1}\end{array}).$$

Finally, using the free-free boundary conditions the normal-mode frequencies of the structured beam are obtained finding the roots of the following equation

6 $${T}_{12}+{T}_{11}{e}^{ik(L-{z}_{M-1})}+{T}_{22}+{T}_{21}{e}^{-ik(L-{z}_{M-1})}=0.$$

More details about the transfer matrix method applied to the elastic beams can be found in refs. ^32^ and^33^.
